# Choledochoduodenostomy Versus Hepaticogastrostomy in Endoscopic Ultrasound-guided Drainage for Malignant Biliary Obstruction: A Meta-analysis and Systematic Review

**DOI:** 10.1097/SLE.0000000000000992

**Published:** 2021-08-31

**Authors:** Kejie Mao, Binbin Hu, Feng Sun, Kaiming Wan

**Affiliations:** Department of Hepatobiliary Surgery, Cixi People’s Hospital, Cixi, Ningbo, People’s Republic of China

**Keywords:** choledochoduodenostomy, hepaticogastrostomy, endoscopic ultrasound, meta-analysis, systematic review

## Abstract

**Objectives:**

This study aimed to estimate the safety and efficacy of endoscopic ultrasound-guided choledochoduodenostomy (EUS-CDS) and endoscopic ultrasound-guided hepaticogastrostomy (EUS-HGS) for malignant biliary obstruction.

**Methods:**

We conducted a literature search using PubMed, Embase, Web of Science, the Cochrane Central Register of Controlled Trials, and ClinicalTrials.gov. Studies that compared EUS-CDS and EUS-HGS were included in this study.

**Results:**

Thirteen studies were eligible for inclusion. The technical [odds ratio (OR): 0.95; 95% confidence interval (CI): 0.51-1.74) and clinical (OR: 1.13; 95%CI: 0.66-1.94) success rates of EUS-CDS were comparable to those of EUS-HGS. However, EUS-CDS had less reintervention (OR: 0.31; 95%CI: 0.16-0.63) and stent obstruction (OR: 0.48; 95%CI: 0.21-0.94) than EUS-HGS. Both groups had similar adverse events (OR: 1.00; 95%CI: 0.70-1.43) and overall survival (hazard ratio: 1.07; 95%CI: 0.58-1.97).

**Conclusions:**

EUS-CDS and EUS-HGS have comparable technical and clinical success rates, adverse events, and overall survival. However, EUS-CDS has less reintervention and stent obstruction.

Endoscopic retrograde cholangiopancreatography (ERCP) with transpapillary stent placement is the standard procedure for unresectable malignant biliary obstruction (MBO).^1^–^3^ Although ERCP drainage has a high success rate, it has a failure rate of 3% to 12% and could cause several complications.^4^–^6^ Endoscopic ultrasonography (EUS), which was first proposed in 2001, has become an alternative treatment method for MBO.^7^ Many trials have been conducted to test the safety and efficacy of endoscopic ultrasound-guided biliary drainage (EUS-BD) for MBO.^8^–^10^ With the development of EUS-BD, several techniques have been developed in studies, including EUS-guided choledochoduodenostomy (EUS-CDS), EUS-guided hepaticogastrostomy (EUS-HGS), EUS-guided rendezvous, and EUS-guided antegrade transpapillary drainage.^11^–^14^ Among these, ESU-CDS and EUS-HGS were the 2 main transluminal methods used in EUS-BD.^15^–^19^ For EUS-CDS, a stent is placed between the common bile duct and duodenum, whereas in EUS-HGS, a stent is inserted from the left hepatic duct into the stomach. Studies have demonstrated variable success rates and adverse events for EUS-CDS and EUS-HGS.^20^–^22^ In recent years, many high-quality studies have been published.^4^,^23^,^24^ However, the safety and efficacy of the 2 methods remain controversial and there is still a lack of consensus on which is better.^25^ Therefore, we included the latest studies and conducted an up-to-date systematic review and meta-analysis to explore and compare the safety and efficacy between EUS-CDS and EUS-HGS.

## METHODS

This systematic review and meta-analysis was conducted in accordance with the Preferred Reporting Items for Systematic Reviews and Meta-Analyses^26^ and Meta-Analysis of Observational Studies in Epidemiology guidelines.^27^ This meta-analysis was registered in the International Prospective Register of Systematic Reviews (PROSPERO) under the number CRD42021231825. Institutional Review Board approval does not apply to this study.

### Search Strategy

A literature search was performed using PubMed, Embase, Web of Science, Cochrane Central Register of Controlled Trials, and ClinicalTrials.gov until December 1, 2020. Specific research equations were developed for each database using the following keywords and/or MeSH terms: “EUS-BD,” “EUS-biliary drainage,” “choledochoduodenostomy,” and “hepaticogastrostomy.” The search was restricted to human patients and English-language full-text articles. Furthermore, we manually reviewed the references of the articles identified after the initial search.

### Inclusion and Exclusion Criteria

Randomized, nonrandomized, and retrospective studies were eligible. In the absence of randomized studies, nonrandomized and retrospective studies were evaluated if they met our inclusion criteria. The exclusion criteria were as follows: (1) review articles, case reports, abstracts, single-arm reports, editorials, and letters to the editor; (2) repeat publication by the same author or agency; and (3) insufficient data on outcome measures.

### Subgroup Analysis

We conducted a subgroup analysis on the studies that used a fully covered self-expandable metal stent (FCSEMS).

### Outcomes of Interest

The primary outcomes of the study were technical and clinical success rates. The secondary outcomes included adverse events, reintervention, and overall survival. To maximize the scope of data collection, the aforementioned outcome measures were defined using the definitions in the original literature. The reintervention was defined as the stent migration, obstruction, or the recurrence jaundice. We compared the reintervention because of stent migration and obstruction between 2 groups.

### Data Extraction and Quality Assessment

Two reviewers independently extracted data, including the author, year of publication, country of origin, study design, samples of intervention, indication of biliary drainage, type of stent, and follow-up time, from the original articles. Conflicts in data abstraction were resolved by consensus and referring to the original article. We assessed the quality of the randomized clinical trials (RCTs) according to the Cochrane Collaboration Handbook,^28^ and non-RCTs were assessed using the criteria of the Newcastle-Ottawa Scale.^29^

### Statistical Analysis

This meta-analysis was performed using Review Manager (RevMan) (version 5.3; Cochrane Informatics and Knowledge Management Department, Nordic Cochrane Centre, Copenhagen, Denmark) and STATA (version 12.0; STATA Corporation, College Station, TX) software. Hazard ratios extrapolated from the Kaplan-Meier curves were calculated for time-to-event outcomes. Odds ratios (ORs) with 95% confidence intervals (CIs) were calculated for categorical variables, whereas standard differences in the means were calculated for continuous variables. The *I* ^2^ index was used as between-study heterogeneity indicator. We used a fixed-effects model where *I* ^2^<50%; otherwise, we used a random-effects model. Where applicable, publication bias was assessed using funnel plots and Egger’s test of asymmetry. Two-tailed *P*-values of <0.05 were considered statistically significant. We assessed the potential for publication bias by visually inspecting a funnel plot asymmetry.

## RESULTS

### Study Selection and Trial Characteristics

**FIGURE 1:**
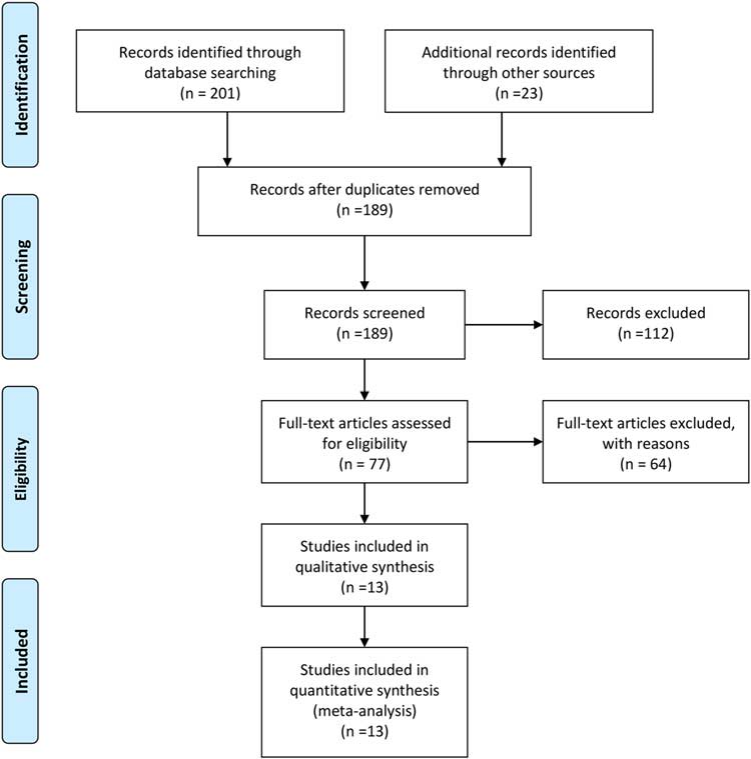
Flow diagram of the published articles that were evaluated in this meta-analysis.

An initial literature search yielded 201 articles through database searching and 23 articles from other sources. After removing duplicates, 189 individual articles remained. Further, 112 records were excluded, followed by screening the title and abstract for various reasons. Finally, 77 articles were identified and underwent a full-text review, after which, 13 trials met the inclusion criteria and were included in this study (Fig. 1).^20^–^24^,^30^–^37^

Thirteen individual articles, including 1 RCT, 3 prospective cohort trials, and 9 retrospective studies, were enrolled in this systematic review and meta-analysis. The studies were conducted between 2012 and 2019. Overall, 759 participants were enrolled in this study, including 359 participants in the EUS-CDS group and 400 participants in the EUS-HGS group. All studies were conducted at various locations such as Brazil, Japan, Korea, China, France, Thailand, and the United States. For studies with insufficient data, we attempted to contact the authors but received no response. The quality of the studies included was tested by two independent authors. A high risk of bias was not detected in any RCT; all had low risks of selection, attrition, and reporting biases. NOS analysis revealed that the studies included were of high quality. The types of stents used included partially covered self-expandable metal stents, FCSEMS, and plastic stents. The characteristics of the studies and quality assessment are summarized in Table 1.

**TABLE 1:** The Characteristics of Included Studies

References	Country	Design	Sample (male)	Age, years (CDS/HGS)	Etiology	Duodenal Invasion	Type of Stent	Follow-up	Quality
Artifon et al^21^	Brazil	RCT	49 (22)	65.77 (15.7)	NR	22	PCSEMS	90 d	Low
				66.25 (14.2)					
Amano et al^20^	Japan	Prospective	20 (11)	73 (45-93)	Pancreatic cancer/bile duct cancer/lung cancer/gastric cancer	17	FCSEMS	NR	NOS-7
							PCSEMS		
Cho et al^22^	Korea	Prospective	54 (29)	64 (29-86)	pancreatic cancer/metastatic cancer/neuroendocrine tumor/cholangiocarcinoma/gall bladder cancer/others	21	PCSEMS	148.5 d (IQR: 79.7-244 d)	NOS-7
				66.3 (44-82)					
Guo et al^30^	China	Retrospective	21 (15)	67 (41-79)	NA	NR	FCSEMS	13 mo (range: 3-21 months)	NOS-6
Kawakubo et al^31^	Japan	Retrospective	64 (35)	72 (66-79)	pancreatic cancer/bile duct cancer/gallbladder cancer/ampullary cancer/metastatic lymph nodes/previous biliary drainage	NR	FCSEMS	103(17-1593)/71 (9-262)	NOS-5
							Plastic stent		
Khashab et al^32^	United States	Retrospective	121 (70)	67.6 (13)	NR	NR	NR	152.2±176.7/151.1±141.1	NOS-5
				63.6 (13.8)					
Kim et al^33^	Korea	Retrospective	13 (9)	69.67 (8.35)	Common bile duct cancer/pancreatic cancer/Klatskin’s tumor/intrahepatic cholangiocarcinoma	2	FCSEMS	Median: 5 months (1-12 months)	NOS-5
				67 (11.17)					
Minaga et al^23^	Japan	Retrospective	47 (25)	73 (41-83)	Pancreatobiliary cancer/others	18	FCSEMS	NR	NOS-6
				72.5 (46-88)					
Ogura et al^34^	Japan	Retrospective	39 (21)	71 (10.7)	Pancreaticobiliary cancer/other	39	FCSEMS	NR	NOS-6
				70 (8.1)					
Park et al^35^	Korea	Prospective	32 (NR)	NA	Pancreatic cancer/hilar cholangiocarcinoma/others	NR	FCSEMS	Mean, 120 d	NOS-7
Poincloux et al^36^	France	Retrospective	96 (NR)	72.2 (10.3)	Pancreatic tumors/cholangiocarcinomas/ampulla of Vater cancers/gallbladder carcinomas/other	25	FCSEMS	280 d (3-775 d)	NOS-6
				69.4 (13.8)					
Prachayakul and Aswakul^37^	Thailand	Retrospective	21 (10)	62.8 (46-84)	Pancreatic cancer/cholangiocarcinoma/gallbladder cancer/others	NR	FCSEMS	NR	NOS-5
Tyberg et al^24^	United States	Retrospective	182 (103)	69.7 (12.8)	Benign/malignant	NR	NR	6 mo, 5.6 mo	NOS-5
				69.9 (12.7)					

CDS indicates choledochoduodenostomy; FCSEMS, fully covered self-expandable metallic stent; HGS, hepaticogastrostomy; IQR, interquartile range; NR, not report; PCSEMS, partially covered self-expandable metal stent; RCT, randomized controlled trial.

### Outcome Measures

#### Primary Outcomes

**FIGURE 2:**
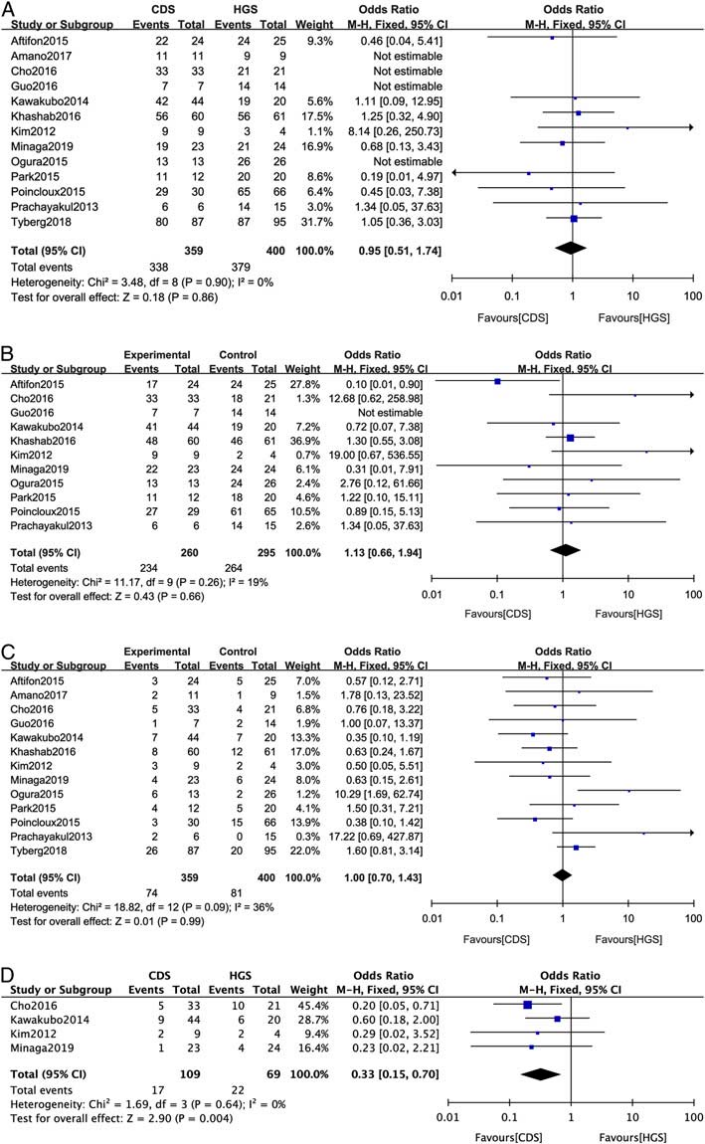
Forest plot of the meta-analysis comparing EUS-CDS and EUS-HGS for (A) technical success, (B) clinical success, (C) adverse events, and (D) reintervention. CI indicates confidence interval; EUS-CDS, endoscopic ultrasound-guided choledochoduodenostomy; EUS-HGS, endoscopic ultrasound-guided hepaticogastrostomy.

In total, 13 studies reported data on technical success. No significant difference was found between the EUS-CDS (338/359) and EUS-HGS (379/400) groups (OR: 0.95; 95%CI: 0.51-1.74; *P*=0.86; *I* ^2^=0%) (Fig. 2A). Eleven studies involving 655 participants provided data on clinical success. The EUS-CDS group has a clinical success rate similar to that of the EUS-HGS group (OR: 1.13; 95%CI: 0.66-1.94; *P*=0.66; *I* ^2=^19%) (Fig. 2B).

### Secondary Outcomes

**FIGURE 3:**
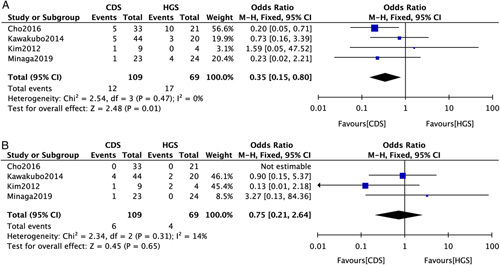
Forest plot of the meta-analysis comparing EUS-CDS and EUS-HGS for (A) reintervention because of stent obstruction, (B) reintervention because of stent migration. CI indicates confidence interval; EUS-CDS, endoscopic ultrasound-guided choledochoduodenostomy; EUS-HGS, endoscopic ultrasound-guided hepaticogastrostomy.

**FIGURE 4:**
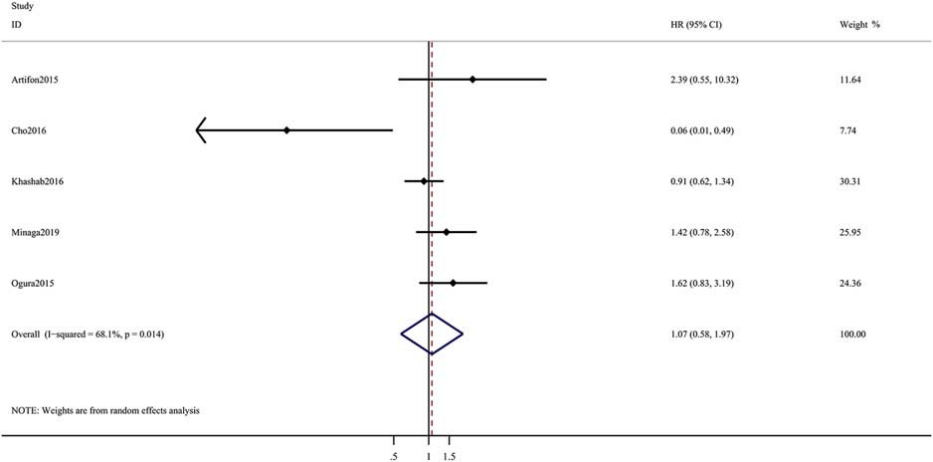
Forest plot of the meta-analysis comparing EUS-CDS and EUS-HGS for overall survival. CI indicates confidence interval; EUS-CDS, endoscopic ultrasound-guided choledochoduodenostomy; EUS-HGS, endoscopic ultrasound-guided hepaticogastrostomy; HR, hazard ratio.

Adverse events in EUS-CDS and EUS-HGS (OR: 1.00; 95%CI: 0.70-1.43; *P*=0.99; *I* ^2=^36%) were similar (Fig. 2C). The subtype analysis of adverse events is shown in Table 2. No difference in cholangitis, bile leakage, pneumoperitoneum, bleeding, and perforation was observed between the 2 groups. Furthermore, no significant difference in stent dysfunction, migration was found between the groups. EUS-CDS has less stent obstruction than EUS-HGS (OR: 0.48; 95%CI: 0.21-0.94; *P*=0.04; *I* ^2=^0%) (Table 2). EUS-CDS was associated with lower rates of reintervention than EUS-HGS (OR: 0.33; 95%CI: 0.15-0.70; *P*=0.004; *I* ^2=^0%) (Fig. 2D). EUS-CDS have less reintervention because of stent obstruction (OR: 0.35; 95%CI: 0.15-0.80; *P*=0.01; *I* ^2=^0%) (Fig. 3A). However, for reintervention because of migration, there was not significantly difference (OR: 0.75; 95%CI: 0.21-2.64; *P*=0.65; *I* ^2=^14%) (Fig. 3B). For overall survival, no significant difference (hazard ratio: 1.07; 95%CI: 0.58-1.97; *P*=0.84; *I* ^2^=68.1%) (Fig. 4) was observed between the groups.

**TABLE 2:** Results of Adverse Events

Outcome of Interest	Studies	Participants	Effect Estimate	*P*	*I* ^2^
All studies					
Cholangitis	7	520	2.17 [0.85, 5.54]	0.10	44%
Bile leakage	8	375	0.71 [0.23, 2.15]	0.54	0%
Pneumoperitoneum	5	374	0.86 [0.22, 3.35]	0.82	0%
Bleeding	6	509	1.61 [0.63, 4.14]	0.32	0%
Perforation	5	455	1.72 [0.46, 6.49]	0.42	0%
Stent dysfunction	8	433	0.55 [0.30, 1.02]	0.06	0%
Stent migration	7	340	0.79 [0.29, 2.14]	0.64	0%
Stent obstruction	6	338	0.48 [0.21, 0.94]	0.04	0%
Studies with FCSEMS					
Cholangitis	3	99	10.29 [1.69, 62.74]	0.01	NS
Bile leakage	5	141	0.44 [0.04, 4.49]	0.49	0%
Pneumoperitoneum	2	135	0.72 [0.03, 18.08]	0.84	NS
Bleeding	1	39	Not estimable	NS	NS
Perforation	1	39	Not estimable	NS	NS
Stent dysfunction	4	177	0.72 [0.25, 2.10]	0.55	0%
Stent migration	3	61	0.73 [0.11, 5.07]	0.75	46%
Stent obstruction	3	99	0.39 [0.09, 1.76]	0.22	0%

FCSEMS indicates fully covered self-expandable metal stent.

### Studies With FCSEMS

**FIGURE 5:**
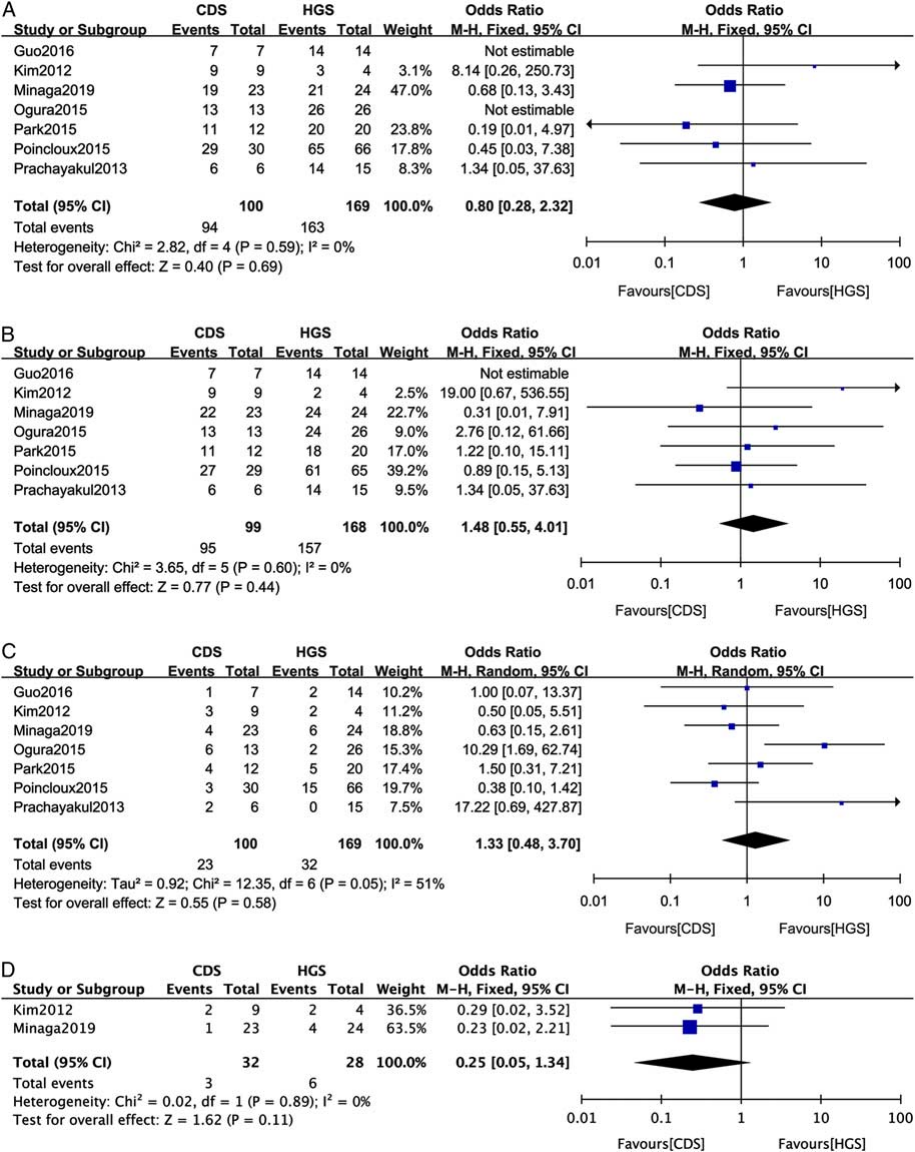
Forest plot of the meta-analysis comparing EUS-CDS and EUS-HGS for (A) technical success, (B) clinical success, (C) adverse events, and (D) reintervention in studies with fully covered self-expandable metal stent. CI indicates confidence interval; EUS-CDS, endoscopic ultrasound-guided choledochoduodenostomy; EUS-HGS, endoscopic ultrasound-guided hepaticogastrostomy.

For studies using FCSEMS, no significant difference was observed in technical success (OR: 0.80; 95%CI: 0.28-2.32; *P*=0.69; *I* ^2=^0%) (Fig. 5A), clinical success (OR: 1.48; 95%CI: 0.55-4.01; *P*=0.44; *I* ^2=^0%) (Fig. 5B), adverse events (OR: 1.33; 95%CI: 0.48-3.70; *P*=0.58; *I* ^2=^51%) (Fig. 5C), and reintervention (OR: 0.25; 95%CI: 0.25-1.34; *P*=0.11; *I* ^2=^0%) (Fig. 5D).

### Sensitivity Analysis

By omitting one study at a time, the influence of a single study on the overall meta-analysis estimate was investigated. Such omission resulted in no significant difference, indicating that our results were statistically reliable.

### Publication Bias

Most graphical funnel plots of the parameters were symmetrical, and Egger test revealed no significant publication bias.

## DISCUSSION

This meta-analysis and systematic review on the largest comparative cohort of studies to date showed that EUS-CDS and EUS-HGS have comparable results in terms of technical and clinical success, adverse events, and overall survival. However, EUS-CDS was associated with less reintervention than EUS-HGS. More high-quality RCTs should be performed.

EUS-BD may be used as an alternative method in patients who are not suitable for ERCP biliary drainage. In terms of treatment success, this meta-analysis demonstrated that EUS-CDS (94.15%) and EUS-HGS (94.75%) had comparable success rates, which were consistent with the literature.^11^,^23^,^24^,^33^,^34^ However, an international multicenter randomized trial published in 2019 revealed that EUS-HGS was associated with a higher technical success rate in patients who underwent EUS-BD for the first time.^23^ The anatomical proximity of the duodenal and extrahepatic bile ducts could decrease the difficulty of EUS-CDS.^7^,^38^ Otherwise, because of its transgastric approach, EUS-HGS can be performed in patients with malignant duodenal obstruction.^3^,^39^ The situation of duodenal obstruction is not explained in detail in the included studies.

Several types of stents were used in the studies included plastic stent, partially covered SEMS, and FCSEMS.^15^,^17^,^40^ We performed a subgroup analysis of the studies that used FCSEMS. Results showed that EUS-CDS and EUS-HGS have similar success rates. In theory, SEMS has a larger diameter and is more conducive to drainage than plastic stents.^3^,^41^,^42^ Kim et al^33^ recommend using EUS-BD with FCSEMS for MBO. The choice between the 2 techniques should be made after determining the degree of dilation of the intrahepatic biliary tree and the ability to access the duodenum.^36^,^43^,^44^ Recently, trials have been conducted to determine the usefulness of lumen-apposing metal stents in EUS-CDS.^19^,^40^ The use of these new devices could result in better success rates and lower rate of occurrence of adverse events.^45^ In the studies included herein, the definition of clinical success was variable. Further studies with more patients and using a unified definition of clinical success are required.

Adverse events occurred after EUS-BD, including cholangitis, bile leakage, pneumoperitoneum, bleeding, and perforation.^13^,^46^–^48^ EUS-CDS and EUS-HGS groups showed similar adverse events in this study. This differs from the results of a study by Hedjoudje et al^11^ This may be related to the number of studies included, the nature of the studies, and the definition of adverse events. One drawback of EUS-BD is bile leakage, which could lead to bile peritonitis; sometimes, it is fatal^17^,^49^ The incidence of bile leakage was 2.68% and 3.17% in the in the EUS-CDS and EUS-HGS groups, respectively. No bile leakage was observed in patients who underwent EUS-CDS with FCSEMS. Several studies have confirmed that FCSEMS could help reduce bile leakage.^22^,^31^,^50^ The present study shows that EUS-CDS can reduce reintervention; however, it should be noted that many factors, including stent migration and obstruction, affect reintervention. Our study showed that EUS-CDS has less stent obstruction. This may be related to the type of stent. Previous studies reported that the stent obstruction ranging between 18% and 46% with the main caused of tumor ingrowth.^51^,^52^ FCSEMS was designed to reduce the occurrence of stent obstruction. The subgroup analysis showed that there was no significant difference in studies with FCSEMS. The reduction of reintervention can theoretically improve the quality of life of such patients. However, there is currently a lack of research on the quality of life. Furthermore, the difference may be related to the definition of reintervention and the incomplete reporting of adverse events in the original study. Because of this conceptual heterogeneity, the pooled estimate should be interpreted cautiously. As for the follow-up results, EUS-CDS and EUS-HGS had similar overall survival. However, chemotherapy data were not detailed in the studies included, which may affect overall survival.

Although our study incorporates most of the original studies that are currently available, some shortcomings remain. First, some results had high heterogeneity owing to the different analyses methods. Second, the definitions of outcomes were variable in the studies included, that is, the use of chemotherapy that may affect overall survival could not be analyzed. The indications of reintervention were variable. Third, inherent heterogeneity bias exists in pooled systematic reviews and meta-analyses. Finally, most studies were retrospective, and some studies used nonrandomized methods, which could lead to selection bias. These limitations warrant caution in the interpretation of the findings of this study. More high-quality studies are required to compare the techniques and refine our results.

In conclusion, EUS-CDS and EUS-HGS have similar efficacy, safety, adverse events, and overall survival for MBO. However, EUS-CD was associated with less reintervention and obstruction. Further RCTs with larger sample sizes are warranted.
